# Cure for Stammering

**Published:** 1884-04-20

**Authors:** 


**Cure for Stammering.**—Reading aloud with the teeth closed, for two hours a day, is said to cure stammering.

